# Integration of an Audiovisual Learning Resource in a Podiatric Medical Infectious Disease Course: Multiple Cohort Pilot Study

**DOI:** 10.2196/55206

**Published:** 2025-02-11

**Authors:** Garrik Hoyt, Chandra Shekhar Bakshi, Paramita Basu

**Affiliations:** 1 Touro University, New York, NY, United States; 2 New York Medical College, New York, NY, United States; 3 New York College of Podiatric Medicine, 53 E 124th St, New York, NY, 10035, United States, 1 212-410-8000

**Keywords:** learning retention, preclinical education, podiatric medical education, audiovisual learning resources, multimedia-based learning resource, animation-supported learning tools, mnemonics, spaced repetition

## Abstract

**Background:**

Improved long-term learning retention leads to higher exam scores and overall course grades, which is crucial for success in preclinical coursework in any podiatric medicine curriculum. Audiovisual mnemonics, in conjunction with text-based materials and an interactive user interface, have been shown to increase memory retention and higher order thinking.

**Objective:**

This pilot study aims to evaluate the effectiveness of integrating web-based multimedia learning resources for improving student engagement and increasing learning retention.

**Methods:**

A quasi-experimental study was conducted with 2 cohorts totaling 158 second-year podiatric medical students. The treatment group had access to Picmonic’s audiovisual resources, while the control group followed traditional instruction methods. Exam scores, final course grades, and user interactions with Picmonic were analyzed. Logistic regression and correlation analyses were conducted to examine the relationships between Picmonic access, performance outcomes, and student engagement.

**Results:**

The treatment group (n=91) had significantly higher average exam scores (*P*<.001) and final course grades (*P*<.001) than the control group (n=67). Effect size for the average final grades (*d*=0.96) indicated the practical significance of these differences. Logistic regression analysis revealed a positive association between Picmonic access with an odds ratio of 2.72 with a 95% confidence interval, indicating that it is positively associated with the likelihood of achieving high final grades. Correlation analysis revealed a positive relationship (*r*=0.25, *P*=.02) between the number of in-video questions answered and students’ final grades. Survey responses reflected increased student engagement, comprehension, and higher user satisfaction (3.71 out of 5 average rating) with the multimedia-based resources compared to traditional instructional resources.

**Conclusions:**

This pilot study underscores the positive impact of animation-supported web-based instruction on preclinical medical education. The treatment group, equipped with Picmonic, exhibited improved learning outcomes, enhanced engagement, and high satisfaction. These results contribute to the discourse on innovative educational methods and highlight the potential of multimedia-based learning resources to enrich medical curricula. Despite certain limitations, this research suggests that animation-supported audiovisual instruction offers a valuable avenue for enhancing student learning experiences in medical education.

## Introduction

Long-term retention is crucial for higher exam scores and overall course grades in preclinical coursework. A recent examination of popular board preparatory resources has provided insight into the different trends that students have experienced in their self-directed learning [1]. Incorporating digital resources appeared to be as effective, if not more so, than regular text-based learning [2]. With greater interest shown in digital learning, curiosity has arisen regarding students’ sentimental value of animation-styled instruction. One study has shown students’ fondness for learning increased with animation instruction as an exciting new way to learn, further increasing permanent learning [3].

Various methods of increasing learning efficiency in medical education have been explored, such as digital recordings, visual mnemonics, and flashcard systems. Many students find the aforementioned methods to be an excellent supplement to the usual textbook-based learning, resulting in higher test scores, particularly within the medical field [4-7]. Enhanced use of digital teaching tools is effective in providing students with basic science information and has shown to be useful in improving their preparation for clerkship [8].

Mnemonics are a commonly used memory technique in medical school. A mnemonic links to well-known knowledge, sometimes invoking humor or emotions [9]. Web-based learning positively impacts information retention and learning efficiency [10,11]. Audiovisual (AV) mnemonics, in conjunction with text-based materials and an interactive user interface, have been shown to increase memory retention and higher order thinking [12,13].

Picmonic [14], a web-based AV learning resource, uses immersive videos, clinical case questions, flashcards, and high-yield notes, as well as picture mnemonics to cover various aspects of the preclinical and clinical practice curriculum [15-17]. A study by Yang et al [12] observed improved student performance in free-recall and paired-matching tests when using Picmonic. Another study by Abdalla et al [13] underscores how important memory and knowledge retention are to a medical student’s grades. Adding AV modalities increases a student’s ability to remember information over an extended period of time. Students who had undergone AV sessions had higher marks on response answer questions, shorter time spent answering questions, and a higher memory consolidation after specific time benchmarks. Further studies of using mnemonics, particularly food eponyms in pathology-related education, have shown relevance in learning and retaining pathology knowledge in addition to being useful for United States Medical Licensing Examination boards preparation, clinical clerkship preparation, and future practice [18].

Other studies have examined the usefulness of incorporating AV instructional tools in various levels of education [19-21], including medical education [1,12,22,23], and found them helpful for improving student engagement and learning experiences [12,22,23]. However, there seems to be a dearth of studies exploring the usage of multimedia web-based learning resources in podiatric medical education. Our goal is to evaluate how integrating a multimedia web-based learning resource affects student engagement and learning retention in a preclinical course. Though there are many online learning resources available for medical students to bolster their learning, we selected the commercially available web-based platform Picmonic due to the shorter length of the videos. Since this resource was integrated into the course in the form of low-stakes assignments with the purpose of serving as a supplementary resource, in addition to the textbook and the instructor-provided materials, it was important to ensure that the videos did not take up too much time.

This pilot study aims to offer insight into the use of tools like Picmonic that uses AV media and mnemonics to supplement traditional learning resources in podiatric medical education. To achieve this goal, we have tried to determine in second-year students in a podiatric medicine program (P) if students who have access to Picmonic, an interactive video-based learning system with mnemonics as an additional supplementary resource (I), show higher course performance and experience better learning retention and engagement with the learning material (O), compared to those with access to textbooks and other instructor-provided course materials only (C), when enrolled in a preclinical infectious disease course in their third semester (T). Course performance and knowledge retention were determined by comparing average final exam scores between a treatment group that had access to Picmonic in addition to textbooks and other instructor-provided material and a control group that relied only on the same textbooks and instructor provided materials. Students’ perception of the usefulness of this platform as a learning resource and engagement with course materials were assessed using a survey instrument and analysis of correlation between the number of in-video questions answered, the number of times the video was watched, and the accuracy of video-embedded quiz attempts.

## Methods

### Study Design

A sample of 158 second-year podiatric medical students enrolled in the Infectious Disease course in the third semester at New York College of Podiatric Medicine (NYCPM) were observed in 2 consecutive cohorts. The cohorts consisted of a control group of 67 students taking the course in 2021 and a treatment group of 91 students taking the course in 2022. Participants in the treatment group used the multimedia web-based learning tool Picmonic as a learning resource, while participants in the control group did not. All students were given the same didactic instruction, textbooks, and other traditional learning resources. The study was conducted as a posttest-only, nonequivalent group, quasi-experimental design [24,25]. Although sample selection was nonrandom, it is assumed that the 2 sample groups are similar in their baseline characteristics as they were both in the same curricular level within the program at the time of taking the course. In addition, the initial knowledge and skill level of the students in the 2 cohorts were determined to be equivalent based on their average cumulative grade point average (GPA) data from the earlier semesters in the program and the average incoming Medical College Admission Test (MCAT) scores and undergraduate GPA. Both cohorts started the course and third semester with similar average standardized test scores, similar mean incoming cumulative GPAs, and were given similar course content and assessments. Other confounders like educator quality and digitalization were addressed by using the same instructors and learning management system for the delivery of course content to both cohorts. There were also no changes made in course instruction, course content, syllabus, grading, or objectives between the 2 cohorts. It was also ensured that contextual confounders such as new academic initiatives or changes in course leadership, program objectives, and fallout from the pandemic did not occur during the period of the study.

In the treatment group, students were given 5% participation credit, which was awarded on the completion of the video-based assignments. A customized playlist of assigned videos (aligned to the lecture topics) curated from the Picmonic video database was created by the instructors to be watched by the students on their own time and answer the embedded quiz questions shown in Multimedia Appendix 1. Each set of assigned videos and quizzes had to be completed before the scheduled in-class lecture on that topic to get credit. Data on the number and frequency of videos watched, multiple attempts at answering video-embedded questions, and quiz accuracy was recorded and monitored using the instructor’s dashboard provided to faculty in the Picmonic platform.

Similarly, in the control group, a 5% participation grade was awarded for active participation in the live discussions held during class time based on prior review of the posted instructional materials and assigned readings from the course textbook to be completed before lecture sessions. The contribution of all other course assessments was weighed identically in both control and treatment cohorts. The instructors, textbooks, lectures, instructor-provided materials, and exams used were kept the same between the 2 cohorts.

Exam scores for the treatment group were collected as posttest observations over the course of the semester. The control group underwent comparable nontreated observations [15,16]. Feedback about user experience was gathered from students enrolled in the treatment group at the end of the course through an electronic web-based semi-structured survey questionnaire modified from Haleem et al [17] consisting of 7 required questions included in the survey instrument as shown in Multimedia Appendix 2. NYCPM’s Institutional Review Board (IRB) granted ethical approval for this study. The students enrolled in the treatment group were sent the survey link, which the student participants voluntarily filled out. The responses to the 7 questions listed under 4 items in the survey instrument were collected, then analyzed and reported as detailed in the Checklist for Reporting Results of Internet E-Surveys (CHERRIES) adapted from [26] and included as Multimedia Appendix 3. Standard strategies appropriate for this type of quasi-experimental study design, consisting of nonrandomized sampling and posttest-only nonequivalent groups, were used for analysis of the data collected [24,25,27].

### Data Analysis

Statistical analysis, data processing, and model fitting were performed in Jupyter Notebook, MATLAB, and Excel. Descriptive statistics, including mean, standard deviation, minimum, maximum, and quartile ranges, were calculated for both the treatment and control group’s exam scores and final grade. Boxplots and histograms display score distributions, central tendency, spread, and outliers. Statistical analysis encompassed Levene tests, Welch *t* tests, and the calculation of Cohen *d* as the effect size [28].

Logistic regression analysis enables us to explore the association between access to Picmonic and receiving a score of 90% or higher through a logistic regression model built using Python’s Statsmodels library for statistical analysis [29]. To fit the model, we used access to Picmonic as a binary predictor (1=access, 0=no access) to predict whether a student achieved a final grade of 90% or higher in the course (1=final grade ≥0.9; 0=final grade <0.9). We obtained the odds ratio from the model by exponentiating the coefficient for the independent variable with the base of the natural logarithm [30]. The log-likelihood ratio was used to evaluate if access to the resource is a relevant predictor of high final grades.

Correlation analysis was performed to determine the strength of the relationship between usage metrics—number of questions answered, videos played, and quiz accuracy —and students’ final grades [30]. We calculated Pearson *r* using a dataset of user interactions with the assigned videos and embedded quizzes on the platform [31].

Survey analysis was conducted using user experience data gathered from students enrolled in the treatment group at the end of the course through an electronic web-based questionnaire sent out by email. Students first answered 4 questions about Picmonic, focusing on information retention, concept understanding, higher test scores, and its usefulness as a learning supplement. Next, students were asked to answer 3 questions regarding their level of satisfaction, frequency of use, and favorite features of the platform—as shown in the Student Experience Survey Instrument in Multimedia Appendix 1. Researchers manually categorized answers to questions regarding their favorite features in Excel. Accordingly, summary statistics were calculated and compared using the data collected from student survey responses.

### Ethical Considerations

This study was approved by NYCPM’s IRB (23575) in May 2022. Informed consent was waived off by the IRB since students agree to the use of unidentifiable education data for research purposes at registration.

As per institutional policy, the IRB approval for this study is a blanket approval provided for all curriculum-related studies, which are undertaken at the college using deidentified aggregate course data rather than individual scores. The original consent or blanket IRB approval covers secondary analysis without additional consent since all incoming new students are required to sign a consent form agreeing to the use of unidentifiable course and education-related data for research purposes at the beginning and is applicable throughout their enrollment in the program.

All students enrolled in the courses that were included in this study were informed about the research and were made aware that the deidentified aggregate course performance data and their feedback would be used to gather data for this pilot study. This information was also reiterated when they were given the survey instruments to record their feedback which was optional for them to fill out.

All data used in this study are course-level aggregate data calculated from score-related data that are anonymized or deidentified.

No compensation was provided for participation in the research study as the courses used are required as part of the podiatric medical curriculum. The students were made aware their feedback would be collected in the form of responses to a survey questionnaire which was optional to complete. Transparency and fairness were ensured by clarifying that the survey instrument was not mandatory and without any consequences for participants who opted out of responding to the questions included in the survey.

## Results

### Statistical Analysis

**Figure 1.**
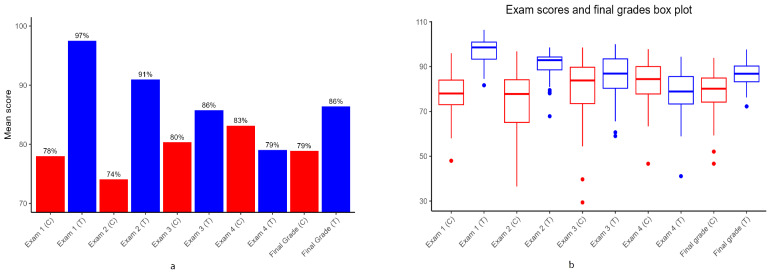
Comparison of student performance in course assessment between treatment group (T) and control group (C) based on (**a**) scaled mean test scores and final course grades from treatment group (having access to Picmonic or AV instruction) and control group (without access to Picmonic or audiovisual instruction) and (**b**) score distributions within each group.

The difference in distribution of exam scores and final grades among the treatment and control groups was visualized using bar graphs (Figure 1A), and box plots (Figure 1B). The summary statistics (Table 1) show the central tendencies using mean exam scores and mean final grade, the spread of the scores using standard deviation, and the shape of the score distributions within each group (Figure 1B), which were used to identify potential differences between the groups. The treatment group had significantly higher average exam scores for most of the course exams and had higher final grades compared to the control group (Figure 1A). The difference in the average scores of the first 2 exams (*P*<.001), the third exam (*P*=.04), and the final course grades (grand total) (*P*<.001) between the 2 groups with and without access to Picmonic was significant. There were also significant differences in variance for exam 1, exam 2, exam 3, and the final grade between the treatment and control groups (Table 2).

**Table 1.** Statistical analysis of student performance in course assessments for treatment and control groups.

Exam	Control, mean (SD)		Treatment, mean (SD)		Mean difference	Welch *t* test *P* value	Pooled SD	Cohen *d*
Exam 1	0.780 (0.098)		0.975 (0.052)		0.195	<.001	0.075	2.398
Exam 2	0.741 (0.143)		0.910 (0.052)		0.169	<.001	0.101	1.486
Exam 3	0.803 (0.133)		0.858 (0.089)		0.054	.005	0.110	0.463
Exam 4	0.832 (0.091)		0.790 (0.092)		−0.041	.006	0.092	−0.449
Final grade	0.789 (0.094)		0.864 (0.049)		0.075	<.001	0.072	0.957

**Table 2.** Analysis of variance of exam scores and final grades between treatment and control groups.

Assessment	Levene test statistic	*P* value	
Exam 1	19.442	<.001^a^	Significant compared to control
Exam 2	52.632	<.001^b^	Significant
Exam 3	4.354	.04	Significant
Exam 4	0.127	.72	Not significant
Final grade	14.652	<.001^c^	Significant

a (1.92×10−5)

b (1.77×10−11)

c (1.87×10−4)

Levene test statistic values for potential differences in variance of exam scores and final grades between the 2 groups revealed significant differences in variance for exam 1, exam 2, exam 3, and the final grade (Table 2). The results of Welch *t* test used to compare the average final exam scores and final grades indicated statistically significant differences between the 2 groups’ first 3 exams and final grades for the course (*P*<.01) (Table 1). Cohen *d* values calculated to quantify the observed differences between the treatment and control groups across all exams and the final grade revealed a large effect size for exam 1 (*d*=2.397), exam 2 (*d*=1.486), and the final grade (*d*=0.957) (Table 1).

### Logistic Regression

A logistic regression analysis between access to Picmonic and the likelihood of achieving a high final course grade of 90 out of 100 (90%) or above resulted in an odds ratio which indicates that, assuming all other factors are constant, students in our study with access to Picmonic were 2.72 times more likely to have received a final grade of 90% or higher and a letter grade of A in the course. The log-likelihood ratio *P* value is .02 (*P<*.05); therefore, we reject the null hypothesis that the base model with only the intercept is better than the model with access to Picmonic used as the predictor (Table 3).

**Table 3.** Regression analysis of the association between access to Picmonic and receiving a high final grade.

Predictor	Coefficient	SE	*z* value	*P* value^a^	Lower CI	Upper CI
Intercept	−1.998	0.377	−5.303	<.001	−2.737	−1.260
Picmonic access	0.971	0.446	2.180	.03	0.098	1.845

a Model log-likelihood ratio *P* value =.02.

### Correlation Analysis

**Figure 2.**
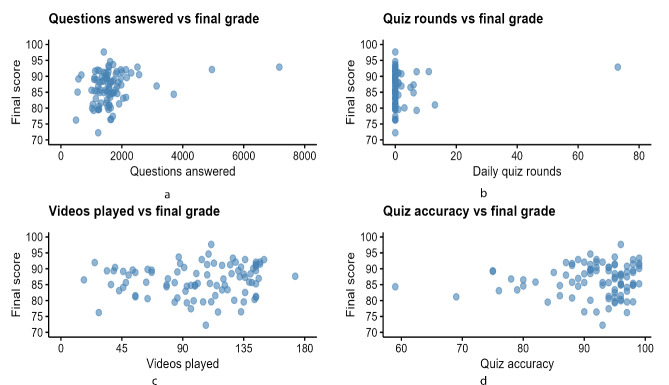
Correlation analysis between final course grades and (**a**) the number of in-video questions answered, (**b**) number of daily quiz round attempts, (**c**) number of times videos watched, and (**d**) overall accuracy of embedded quizzes.

Pearson correlation coefficient calculated to explore the relationship between final course grade and various usage statistics (number of videos played, questions answered, and quiz accuracy) show that students in the treatment group who answered more questions on the platform tended to get a higher final score (*r*=0.25, *P*=.02) (Table 4 and Figure 2).

**Table 4.** Analysis of correlation between final grades and platform usage metrics.

Final grade with:	Pearson *r*	*P* value
Questions answered	0.247965674	.02
Daily quiz rounds	0.124515676	.24
Videos played	0.09980258	.35
Quiz accuracy	0.074970912	.48

### Survey Analysis

**Figure 3.**
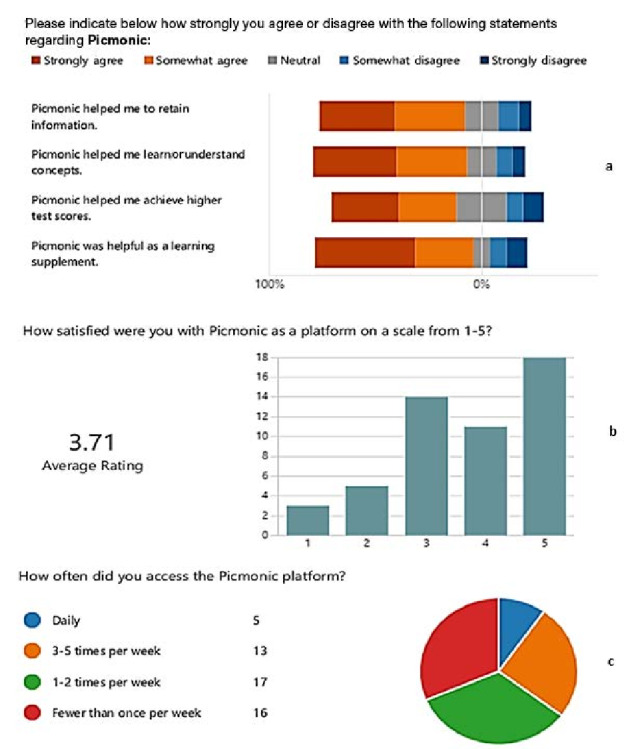
Analysis of student experience feedback data. Student experience survey insights and summary of qualitative open-ended student feedback data showing (**a**) perceived effectiveness of Picmonic on various learning outcomes and retention, (**b**) user satisfaction level, and (**c**) frequency of use.

The response rate for item 1 of the survey instrument, which consisted of 4 questions about the students’ perceived usefulness of Picmonic, was 73% (66 out of 91). In this item, students indicated strong agreement regarding Picmonic’s positive impact on information retention, concept understanding, higher test scores, and usefulness as a supplementary learning tool (Figure 3A). In item number 4, students also reported an average satisfaction rating of 3.71 out of 5 (Figure 3B). Out of 53 students who responded to item number 2 in the survey questionnaire, 36 accessed Picmonic at least once a week—predominantly 1‐2 times per week (Figure 3C). In item number 3, 50 out of 91 students responded to the open-ended questions regarding user experience or preferences about their favorite feature of Picmonic with some choosing more than 1 feature. The number of times each feature was reported as preferred is listed and compared in Table 5. Noteworthy features of Picmonic highlighted by students included videos, quizzes and questions, mnemonics, and content (Table 5).

**Table 5.** Comparative analysis of student feedback performed on open-ended questions regarding user experience or preferences.

	Feedback (N=50), n (%)
Videos	15 (30)
Quizzes and questions	9 (18)
Mnemonics	9 (18)
Content	9 (18)
Images	6 (12)
User interface	4 (8)
General	2 (4)

## Discussion

### Principal Findings

In our study, we observed that students with access to the multimedia and mnemonic-based AV learning resource scored higher on most exams, had higher final grades, and were more likely to receive a final grade of 90% or higher. The resulting effect sizes for the treatment group are large enough to be meaningful in the real world; however, other factors may have contributed to the observed effect size. Students in the treatment group who answered more questions on the platform tended to get a higher final score. Most students used Picmonic at least once a week to learn the course content. The lower response rate on this item asking about the frequency of accessing Picmonic in a week could be due to the inability of participants to remember their frequency of access at the time of completing the survey several weeks after the completion of the course and semester. Students had positive opinions regarding Picmonic’s platform user experience and its effectiveness in helping them retain information, learn and understand concepts, and achieve higher test scores. In this course, the students reported the videos to be their most preferred feature, followed by mnemonics and self-assessment quiz questions associated with the videos, which allowed them to test their knowledge both during and after watching the assigned videos on their own time with unlimited attempts. In this context, it is important to note that though students generally prefer to watch videos on their own rather than attending class, the 2 groups were treated equally since both the reading assignments (for the control group) and the Picmonic-based video assignments (for the treatment group) were assigned to be completed outside of class on their own time. Additionally, the students in the treatment group were assigned to watch the videos and complete the video-embedded quizzes within a specific time frame to mimic the reading assignments given to the control group. To receive the 5% participation grade, the treatment group had to complete the Picmonic video assignments within the instructor-provided deadline corresponding to the weekly topical schedule, which is similar to that of the control group, rather than watching the assigned videos at their own pace throughout the semester.

### Comparison With Previous Research

Our experimental results indicate that students with access to the multimedia-based AV mnemonic learning resource scored higher on most exams, had higher final grades, and were more likely to receive a final grade of 90% or higher. The difference between the grades achieved by the 2 groups is large enough to be meaningful in the real world. These findings are consistent with previous studies that have demonstrated the effectiveness of AV mnemonics and web-based learning tools in enhancing memory retention and learning outcomes in medical education [12,16].

Yang et al [12] observed improved student performance in free-recall and paired-matching tests when using an earlier version of the multimedia-based learning platform that we have used here that was released almost 10 years ago, while the current version that we have used has newer, redesigned, more impactful, and shorter videos, though still based on the same type of picture mnemonics and principles of spaced repetition and visual learning. The currently available version that was used in this study also has improved dashboard features and assessment capabilities compared to the older version. Abdalla et al [13] also found that students who had undergone AV sessions had higher marks on response answer questions, shorter time spent answering questions, and higher memory consolidation after specific time benchmarks. These studies underscore the importance of memory and knowledge retention in medical students’ academic performance.

Results examining user interaction with the resource showed that the more questions a student answered on a multimedia-based AV learning platform (like Picmonic) using spaced repetition and mnemonics, the higher their grade tended to be. This finding aligns with research highlighting the benefits of interactive user interfaces and spaced repetition in increasing memory retention and higher order thinking [12].

Survey responses indicated that students found the resource useful for learning concepts, retaining information, and achieving higher test scores. This is consistent with previous research demonstrating increased student engagement, comprehension, and satisfaction with multimedia-based resources compared to traditional instructional methods [15,18-20]. Studies by Tackett et al [23] examined student engagement with commercially produced medical education videos incorporated into a preclinical course and also found the videos to be helpful for student learning and improved students’ experiences.

Overall, our findings contribute to the growing body of evidence supporting the integration of multimedia-based learning resources and AV mnemonics in medical education curricula to enhance student learning experiences and outcomes [7,8,12,16,17].

### Limitations

While this investigation showed promising initial findings, we recognize that the study design limits its ability to make unequivocal causal inferences about the impact of the multimedia tool alone on the outcomes. The sample size is relatively small, and the lack of randomization in the study design may limit the generalizability of the findings. The quasi-experimental design with nonrandom assignment to treatment and control groups restricts the establishment of a cause-and-effect relationship [24], which could potentially affect the internal validity of the study and its ability to accurately infer whether the change in outcomes was caused by the intervention. Without randomized selection of the 2 groups, we cannot rule out potential unmeasured differences due to systematic differences in sample selection [23]. The study only examined the effect of the implementation of the intervention in 1 preclinical infectious disease course, limiting assessment of the tool’s effectiveness. Additionally, the 5% participation credit component was implemented differently for the control (class participation) versus treatment (video watching) groups, which could impact effort levels.

The 2 most likely influential unmeasured confounding variables in our study are potential differences in the overall academic aptitude of the students in each sample [13], as well as potential differences in the amount of time spent with study materials between the 2 groups due to the spaced repetition provided in the Picmonic platform, which could impact the results. The potential difference in student aptitude between the 2 groups should be somewhat mitigated by the fact that both groups were 2nd-year medical students at the same college when being tested. Additionally, the 2 groups had similar average standardized test scores and mean incoming GPAs at the beginning of the semester. The absence of pretest observations comparing the treatment group to the control group makes it difficult to know whether any differences between the 2 groups could potentially be attributed to pre-existing factors rather than the multimedia learning tool alone [14].

Since this study was done on a very limited number of students and involved only 1 course focused on the topic of preclinical medical microbiology and infectious diseases, this effect may not be generalized for all topics or types of courses. This course is heavily dependent on memorization and recall due to the nature of the topics covered, which may also contribute to the impact of the integration of visual learning with mnemonics and spaced repetition and therefore may not be equally applicable in another course that does not require extensive memorization.

### Conclusions

Our study shows strong agreement amongst students that Picmonic helped them achieve key learning outcomes. Usage data revealed a positive relationship between final grades and students’ usage of platform features; the number of in-video questions answered had a stronger correlation than the number of times videos were watched or the accuracy of topic-associated quiz attempts. Students that were given access to Picmonic did better on exams; however, it must be noted that due to the nonrandomized sampling process, posttest-only study design, limited implementation, and small sample size, it is difficult to conclude whether the difference was due solely to the integration of the new tool. The improved course grades and test scores in the treatment group may have been due to the inherent confounding factors like comprehension and retention skills or increased contact time with the course topics provided by the platform features. Our pilot study focused on the integration of Picmonic, a multimedia-based learning resource, in only 1 course, but implementing it across more courses over multiple semesters would strengthen the assessment of the tool’s effectiveness. Despite its limitations, this study provides insight into the potential benefits of integrating multimedia learning resources in podiatric medical education. However, a larger study that implements this type of learning resource on a larger scale, and in more preclinical and clinical courses throughout the curriculum, is needed to further analyze its effectiveness in the podiatric medical curriculum.

## Supplementary material

10.2196/55206 Multimedia Appendix 1 Example of the playlist of Picmonic videos and embedded quiz questions on Gram positive bacilli assigned to the treatment group students.

10.2196/55206 Multimedia Appendix 2 Student user experience survey instrument.

10.2196/55206 Multimedia Appendix 3 Checklist for Reporting Results of Internet E-Surveys (CHERRIES).

## Abbreviations

AV: audiovisual
CHERRIES: Checklist for Reporting Results of Internet E-Surveys
GPA: grade point average
IRB: Institutional Review Board
MCAT: Medical College Admission Test
NYCPM: New York College of Podiatric Medicine
