# Supplementary figures and images for: Integration of an Audiovisual Learning Resource in a Podiatric Medical Infectious Disease Course: Multiple Cohort Pilot Study
Source: JMIR Med Educ. 2025 Feb 11;11:e55206. doi: 10.2196/55206 (PMC11835597; doi:10.2196/55206)

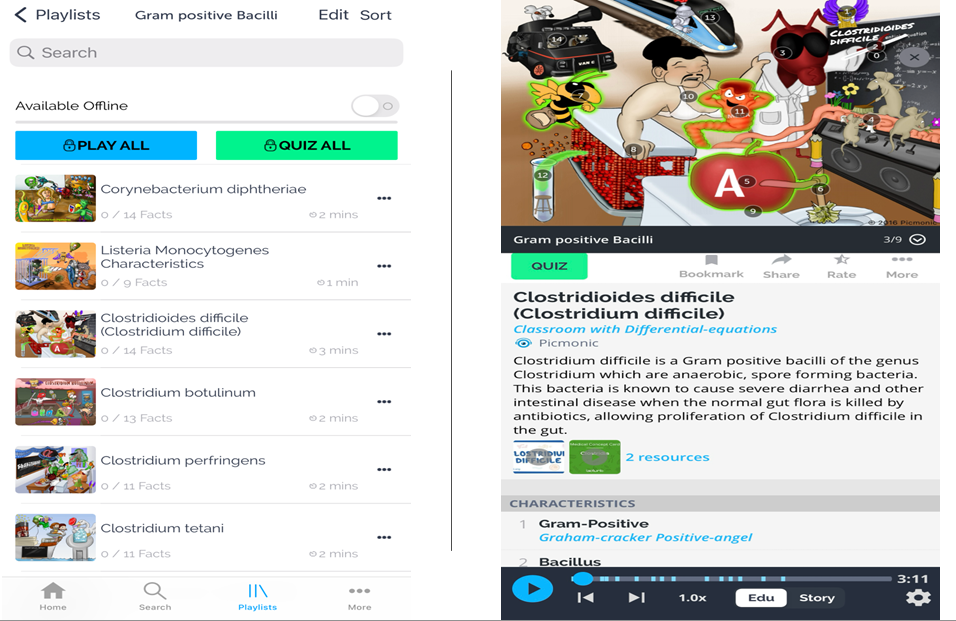

Supplement: Multimedia Appendix 1 [file mededu-v11-e55206-s001.png]

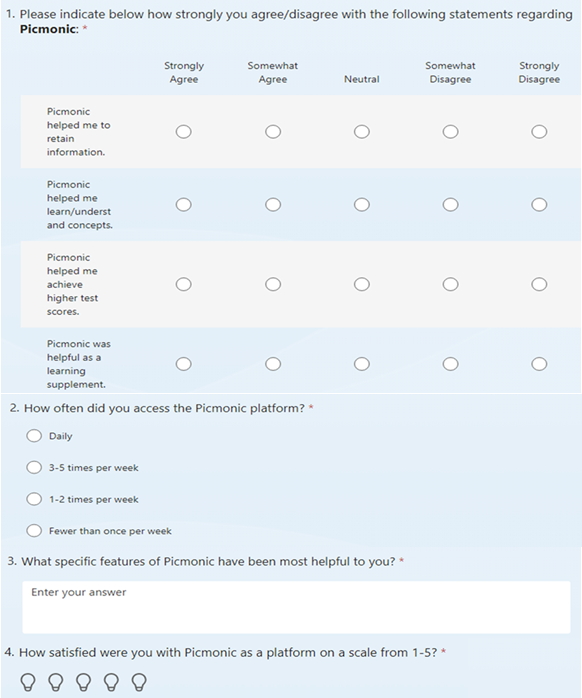

Supplement: Multimedia Appendix 2 [file mededu-v11-e55206-s002.png]
