# Supplementary material for: Integration of an Audiovisual Learning Resource in a Podiatric Medical Infectious Disease Course: Multiple Cohort Pilot Study
Source: JMIR Med Educ. 2025 Feb 11;11:e55206. doi: 10.2196/55206 (PMC11835597; doi:10.2196/55206)
Supplement: Multimedia Appendix 3 [file mededu-v11-e55206-s003.pdf]

**Checklist for Reporting Results of Internet E-Surveys (CHERRIES)**

| Item category | Explanation |
|---|---|
| Design | The study involved a convenience sample. Eligibility criteria included female, 18 years of age or older, and University of Arizona undergraduate. |
| IRB | **Approval.** The study has been approved by the NEW York College of Podiatric Medicine IRB.<br><br>**Informed consent.** Participants were informed of the purpose of the survey, anonymity, confidentiality, and voluntary principles before responding. In the email sent to student participants was the length of time to complete the survey, principal investigator name, and data storage information.<br><br>**Data protection.** No personally identifying information was collected. |
| Development and pre-testing | The survey was developed through reviewing the literature and pilot-testing the survey during the end of the semester after completion of final exam and release of grades. |
| Recruitment process | **Survey type.** The data was collected using an open survey.<br><br>**Contact mode.** Initial contact with participants was made on the Internet. An email was sent to all students enrolled in the Treatment group inviting them to consider responding to the survey.<br><br>**Advertising the survey.** The principal investigator (Dr. Paramita Basu) sent the email invitation to participate to these students through the course listserv. The link to the survey was embedded in the email invitation to participate. |
| Survey administration | **Web/E-mail.** The survey was published on an online questionnaire platform. The survey link was sent via email through the course listserv in the Learning Management System.<br>Data was entered automatically when participants responded to the questions.<br><br>**Context.** The commercial platform specializes in publishing online questionnaires. The authors only used it for data collection.<br>At the time of the study, Dr. Basu (co- author) was the Director of and coordinator of the course, responsible for design of the course, its contents, materials, resources, and assessments. Both Dr. Basu and Dr. Bakshi (co-author) were instructors responsible for content delivery and assessments. Mr. Hoyt (author) was responsible for design, creation, testing and delivery of the survey with Dr. Basu and also collection and analysis of responses. |

| | |
|---|---|
| | **Mandatory/voluntary.** The survey was voluntary.<br><br>**Incentives.** None.<br><br>**Time/Date.** 2022<br><br>**Randomization of items or questionnaire.** N/A<br><br>**Adaptive questioning.** N/A<br><br>**Number of items.** There were 4 items with one item consisting of 4 sub-questions.<br><br>**Number of screens.** four screens.<br><br>**Completeness check.** Manual completeness checks were done during the data analysis phase.<br><br>**Review step.** Participants could use a Back button. |
| Response rates | **Unique site visitor.** N/A<br><br>**View rate.** N/A<br><br>**Participation rate.** The overall participation rate was not calculated since the number of responses was different for each item number.<br><br>**Completion rate.** The completion rate was not calculated since some participants did not respond to all the items. |
| Preventing multiple entries | **Cookies used.** Cookies were not used.<br><br>**IP check.** IP addresses were not collected since the subjects are college students, they usually use the campus network, so the IP address may be repeated.<br><br>**Log file analysis.** The study did not include a log file analysis.<br><br>**Registration.** N/A |
| Analysis | **Handling of incomplete surveys.** Completed surveys were analyzed. For the small number of surveys with missing responses, only the responses to completed items were analyzed.<br><br>**Questionnaires submitted with an atypical timestamp.** N/A<br><br>**Statistical correction.** N/A |
